# Analysis of Drug Effects on Primary Human Coronary Artery Endothelial Cells Activated by Serum Amyloid A

**DOI:** 10.1155/2018/8237209

**Published:** 2018-02-13

**Authors:** K. Lakota, D. Hrušovar, M. Ogrič, K. Mrak-Poljšak, S. Čučnik, M. Tomšič, B. Božič, P. Žigon, S. Sodin-Semrl

**Affiliations:** ^1^Department of Rheumatology, University Medical Centre Ljubljana, SI-1000 Ljubljana, Slovenia; ^2^Faculty of Mathematics, Natural Science and Information Technologies, University of Primorska, SI-6000 Koper, Slovenia; ^3^Blood Transfusion Center of Slovenia, Tissue Typing Centre, SI-1000 Ljubljana, Slovenia; ^4^Faculty of Pharmacy, University of Ljubljana, SI-1000 Ljubljana, Slovenia; ^5^Faculty of Medicine, University of Ljubljana, SI-1000 Ljubljana, Slovenia

## Abstract

**Background:**

RA patients have a higher incidence of cardiovascular diseases compared to the general population. Serum amyloid A (SAA) is an acute-phase protein, upregulated in sera of RA patients.

**Aim:**

To determine the effects of medications on SAA-stimulated human coronary artery endothelial cells (HCAEC).

**Methods:**

HCAEC were preincubated for 2 h with medications from sterile ampules (dexamethasone, methotrexate, certolizumab pegol, and etanercept), dissolved in medium (captopril) or DMSO (etoricoxib, rosiglitazone, meloxicam, fluvastatin, and diclofenac). Human recombinant apo-SAA was used to stimulate HCAEC at a final 1000 nM concentration for 24 hours. IL-6, IL-8, sVCAM-1, and PAI-1 were measured by ELISA. The number of viable cells was determined colorimetrically.

**Results:**

SAA-stimulated levels of released IL-6, IL-8, and sVCAM-1 from HCAEC were significantly attenuated by methotrexate, fluvastatin, and etoricoxib. Both certolizumab pegol and etanercept significantly decreased PAI-1 by an average of 43%. Rosiglitazone significantly inhibited sVCAM-1 by 58%.

**Conclusion:**

We observed marked influence of fluvastatin on lowering cytokine production in SAA-activated HCAEC. Methotrexate showed strong beneficial effects for lowering released Il-6, IL-8, and sVCAM-1. Interesting duality was observed for NSAIDs, with meloxicam exhibiting opposite-trend effects from diclofenac and etoricoxib. This represents unique insight into specific responsiveness of inflammatory-driven HCAEC relevant to atherosclerosis.

## 1. Background

A healthy endothelium provides for an antiadhesive/antithrombogenic surface, which can prevent the development of atherosclerosis and thrombosis. Systemic autoimmune diseases, such as rheumatoid arthritis (RA), exhibit accelerated atherosclerosis (AS) [1–4] as a consequence of endothelial dysfunction, leading to higher incidence of cardiovascular (CV) disease (at least 2-fold enhanced CV risk) and premature and higher mortality [5, 6]. The pivotal role of inflammation in the development of AS and amplification of CV risk in RA has been extensively and well documented [7–10].

Inflammation mediates all stages of atherosclerotic CV events, from preclinical initiation to thrombotic complications of AS [11]. Serum amyloid A (SAA), a major acute-phase protein and inflammatory marker, has long been implicated as a predictor of clinical progression and outcome in RA [12] and a predictor of coronary artery disease, CV outcome [13], and early mortality in acute coronary syndromes [14]. SAA was shown to exhibit causal properties in AS, as a consequence of endothelial dysfunction (elevating tissue factor, as well as a variety of cytokines/chemokines) and early lesions (biglycan synthesis) [15] to plaque destabilization by inducing matrix metalloproteinases [16]. The first report in 2007, on SAA-stimulated human coronary artery endothelial cells (HCAEC), exhibited a substantial and significantly higher induction of released IL-6 protein and mRNA levels as compared to HUVEC [17], as well as increased responsiveness to IL-1*β* [18]. SAA dose-dependently increased IL-6 protein levels in HCAEC, to a much larger extent than in HUVEC (4-fold higher at a concentration of 1000 nM SAA). These changes were not only confirmed by IL-6 mRNA expression levels but also showed larger changes (>20-fold), judging by densitometry [17]. It is unclear, however, how drugs used routinely in rheumatology for treating RA and other chronic diseases can affect HCAEC, in the presence of SAA.

A wide variety of drugs from different groups of functionality was tested in our cellular model, namely, (a) a glucocorticoid (GC), for example, dexamethasone; (b) disease-modifying antirheumatic drugs (DMARDs), for example, methotrexate; (c) biologicals and anti-TNF*α* inhibitors, for example, etanercept and certolizumab pegol; (d) an angiotensin-converting enzyme (ACE) inhibitor, for example, captopril; (e) an antilipemic agent, for example, fluvastatin; (f) an antidiabetic thiazolidinedione (TZD), for example, rosiglitazone; and (g) three nonsteroidal anti-inflammatory drugs (NSAIDs), for example, diclofenac, meloxicam, and etoricoxib.

Dexamethasone is a synthetic GC that binds to cytosolic glucocorticoid receptors, translocates to the nucleus, and physically interacts with NF-*κ*B and AP-1 thereby affecting expression of IL-1, IL-6, TNF*α*, and VCAM, among others, and attenuating the inflammatory response [19, 20].

Methotrexate (MTX) is an antimetabolite used in low doses for treatment of autoimmune diseases. It is the most widely used classic DMARD, inhibiting dihydrofolate reductase and purine synthesis, acting as anti-inflammatory by causing adenosine release and signaling through adenosine G-protein-coupled receptors [21]. MTX reduced levels of proinflammatory cytokines in patients on one hand and increased anti-inflammatory cytokines on the other [22].

TNF*α* is a cytokine, central for the development of the inflammatory response in RA [23], present in soluble (17 kDa) and precursor membrane-bound form (26 kDa) found also on the endothelium [24, 25]. Clinical trials using anti-TNF*α* biologicals, such as etanercept and certolizumab pegol, to treat rheumatic diseases started in the mid-1990s [26] and today represent an important part of RA patient therapy, especially for those who fail to respond to traditional nonbiological DMARDs.

Captopril was the first marketed ACE inhibitor. ACE is mainly expressed on the endothelium surface [27, 28] with oxLDL shown to induce ACE in HCAEC [29]. This class of drugs affects the renin-angiotensin-aldosterone system by cleaving angiotensin I in angiotensin II, increasing water retention and vasoconstriction, making captopril an antihypertensive agent. ACE also degraded bradykinin, a potent vasodilator [30, 31], exhibited anti-inflammatory actions, affected scavenging reactive oxygen species, and influenced prostaglandin production, as well as levels of certain inflammatory cytokines [32, 33].

Statins were first marketed in 1987 [34], with the main indication for hypercholesterolemia and ischemic heart disease prevention. Their mechanism was shown to go through inhibition of liver HMG-CoA reductase, influencing cholesterol synthesis by producing mevalonate and lowering low-density lipoprotein (LDL). Studies also reported beneficial effects on C-reactive protein (CRP) lowering (as reviewed by Liao [35]), and specifically, the JUPITER study pointed out that subjects with increased CRP without hypercholesterolemia could benefit from statin therapy, regardless of LDL levels [36]. Fluvastatin is a synthetic statin, shown to reduce coronary events when started after percutaneous coronary intervention [37].

Thiazolidinediones, such as rosiglitazone, are exogenous agonists of peroxisome proliferator-activated receptor *γ* (PPAR*γ*), a nuclear receptor acting as a transcription factor also found present in atherosclerotic plaques. Rosiglitazone improved endothelial dysfunction; decreased CRP, SAA, and E-selectin [38]; and was shown to promote generation of the anti-inflammatory lipid mediator 15-epi lipoxin A_4_ [39].

NSAIDs are widely used for their anti-inflammatory and analgesic properties in rheumatic diseases, promoting inhibition of COX-2 activity and prostaglandin synthesis as the main mechanisms of action. In addition, they were reported to inhibit NF-*κ*B [40] and activate PPARs [41]. However, different NSAIDs showed differential modes of activity; for example, diclofenac, a derivative of acetic acid, acted similarly to COX-2 selective inhibitors in increasing risk of myocardial infarction (MI) [42], as was the case for all NSAIDs depending on dose administered, as they all inhibit COX-2 enzyme activity [6, 43]. Because it is unclear how NSAIDs affect the coronary artery endothelium, we set out to compare three different NSAIDs, specifically potent diclofenac, highly selective COX-2 inhibitor etoricoxib, and enolic acid-derived meloxicam on stimulated HCAEC.

Besides traditional risk factors, therapy might influence both the development and even more importantly, the regression of AS [5, 6]. Thus, the main aim of our study was to determine the impact of the aforementioned drugs used for therapy of systemic autoimmune diseases, such as RA, on inflammatory responses of SAA-activated HCAEC, suggesting their effects on the coronary artery endothelium.

## 2. Materials and Methods

### 2.1. Cell Culture

Human coronary artery endothelial cells (HCAEC) were purchased from Cambrex BioScience (Walkersville, Maryland, USA). Cells were plated into 6-well plates (TPP, Trasadigen, Switzerland) at 37°C in a humidified atmosphere at 5% CO_2_ and grown in EGM-2M medium containing 5% fetal bovine serum, following the manufacturer's instructions (Cambrex BioScience, Walkersville, MD, USA).

### 2.2. Materials

Lyophilized human recombinant SAA1/2 (hrSAA1/2) (Peprotech EC Ltd., London, UK) was spun down and reconstituted according to the manufacturer's instructions in cell culture-grade sterile water to a stock concentration of 1 *μ*g/*μ*l and stored until used at −20°C or −80°C.

The following medications were tested: (a) dexamethasone (Krka, Slovenia; stock 4 mg/ml), final concentration 5 *μ*M; (b) methotrexate (Medac, Germany; stock 10 mg/ml), final concentration 1 *μ*M; (c) certolizumab pegol (UCB Pharma, Belgium; stock 200 mg/ml), final concentration 100 *μ*g/ml; (d) etanercept (Pfizer, UK; stock 50 mg/ml), final concentration 100 *μ*g/ml; (e) captopril (Krka, Slovenia; stock 25 mg), final concentration 10 *μ*M, dissolved in medium; (f) fluvastatin sodium (Novartis, Germany; 40 mg), final concentration 10 *μ*M, dissolved in DMSO; (g) rosiglitazone (Cayman Chemical, USA; stock 10 mg/ml), final concentration 30 *μ*M, dissolved in DMSO; (h) diclofenac sodium (Krka, Slovenia; 75 mg), final concentration 10 *μ*M, dissolved in DMSO; (i) meloxicam (Boehringer Ingelheim, Germany; 15 mg), final concentration 100 *μ*M, dissolved in DMSO; and (j) etoricoxib (MSD, Netherlands; 90 mg) final concentration 100 *μ*M, dissolved in DMSO.

### 2.3. HCAEC Treatments

**Scheme 1:**
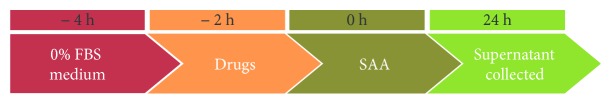
Timeline protocol.

HCAEC at passage 5, grown to confluency in 6-well plates, were incubated in serum-free media for 2 hours prior to experiments. Preincubation was performed for 2 hours with the specific medications from sterile ampules or resuspended, at above indicated final concentrations, followed by the addition of SAA1/2 to stimulate HCAEC at a final 1000 nM concentration for 24 h (Scheme 1), and supernatants were collected, aliquoted, and stored at −20°C until tested.

### 2.4. Enzyme-Linked Immunosorbent Assay

Protein levels of IL-6, IL-8, PAI-I, and sVCAM-1 (all Invitrogen, Frederick, MD, USA) were measured in cell culture supernatants using ELISA.

The assays were performed in duplicates according to the manufacturer's instructions. Briefly, samples were diluted with standard diluent buffer 1 : 50 for IL-6, 1 : 2 for sVCAM-1, 1 : 50 for IL-8, and 1 : 80 for PAI-1 ELISA. In all ELISAs, biotin-labeled conjugates were incubated with samples for 2 hours and, after washing, incubated with streptavidin-horseradish peroxidase enzyme. Tetramethylbenzidine was used as a substrate, and after the reaction was stopped, absorbance was measured at 450 nm with a Sunrise Tecan microplate absorbance reader (Tecan, Groening, Austria). The concentrations of analytes were calculated from standard curves and multiplied by the dilution factor.

In order to compare the results of many cell culture experiments, we had to normalize the data—so a response in a well with the SAA treatment was taken as 1 in each experiment and responses in all other wells were calculated accordingly.

### 2.5. Viability

The number of viable cells was determined colorimetrically (CellTiter MTS assay, Promega). Cell toxicity and cell viability were assessed by cell morphology and with CellTiter 96 Aqueous One Solution Reagent (Promega, Madison, WI, USA), respectively. The viability assay was modified for use with adherent cells. After completion of treatments in 6-well plates, cells were washed with PBS and 200 *μ*l of fresh serum-free medium was added together with 20 *μ*l of reagent. Following 20 minutes, 100 *μ*l of medium was transferred to a 96-well plate and absorbance read at 490 nm.

### 2.6. Statistical Analysis

All experiments were repeated at least in biological triplicate. Data are presented as mean ± standard deviation (SD). Means were compared among the various treated and control groups using Student's *t*-test. *p* values of <0.05 were accepted as statistically significant, unless otherwise stated.

## 3. Results

**Figure 1:**
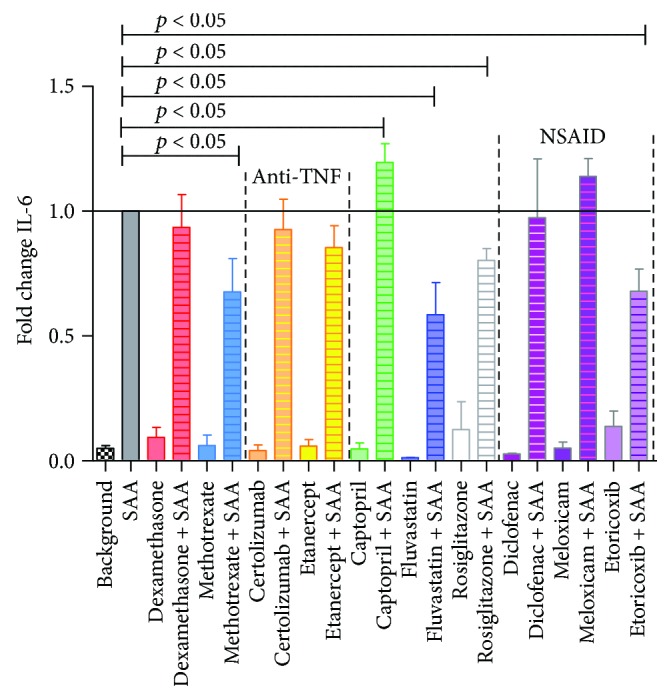


**Figure 2:**
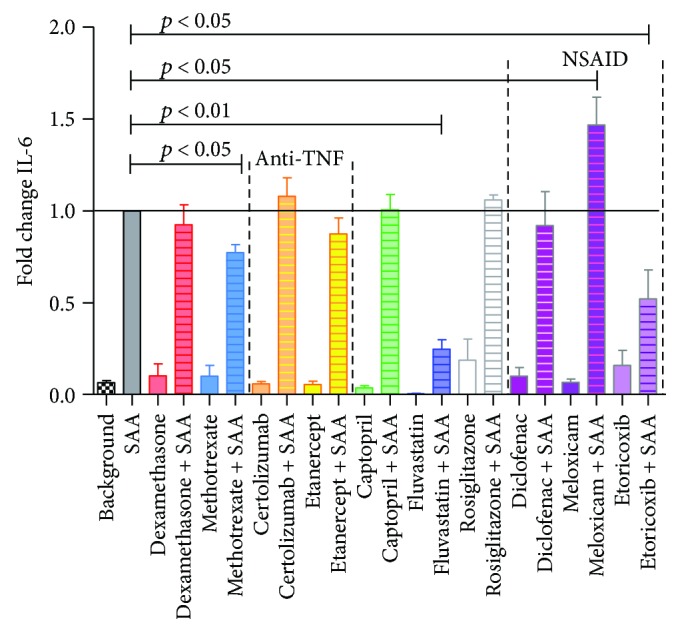


In order to determine the inflammatory response of HCAEC, stimulated for 24 h with pathological concentrations of SAA (1000 nM), in the presence and absence of drugs, released IL-6 and IL-8 protein levels were measured (Figures 1 and 2). Of all tested drugs, only captopril treatment significantly increased IL-6 in SAA-stimulated HCAEC (by 19%), while methotrexate and etoricoxib reduced IL-6 levels to 67%, with fluvastatin exhibiting the largest inhibition, down to 58% of initial SAA stimulatory levels. The three NSAIDs showed different modes of activity, with meloxicam increasing IL-6 (by 14%), diclofenac not affecting IL-6 levels, and etoricoxib significantly decreasing IL-6 levels (to 67%) (Figure 1).

Similarly, IL-8 protein production exhibited a marked, significant inhibition in the presence of fluvastatin (down to 24% of SAA-treated HCAEC) followed by methotrexate (to 77%) and etoricoxib (to 52%). On the other hand, meloxicam increased IL-8 (by 46%), similar to IL-6 (Figure 2).

**Figure 3:**
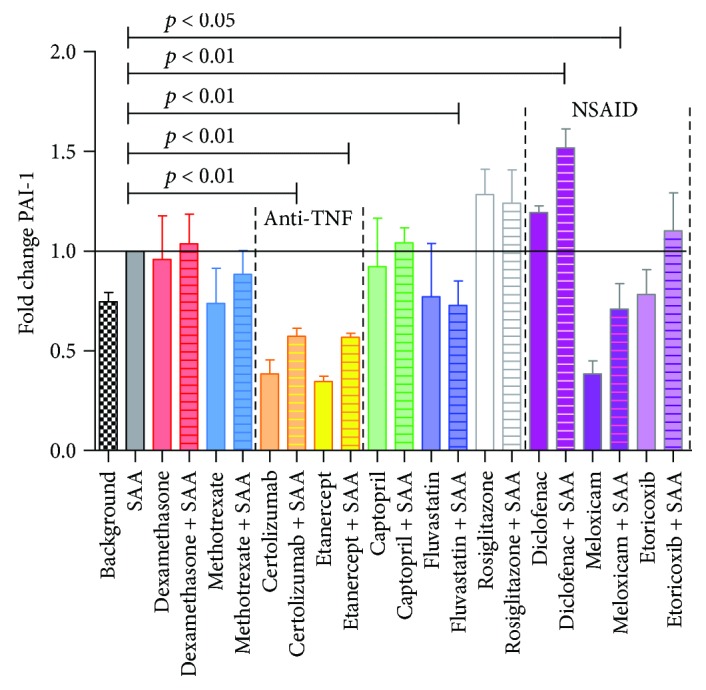


Since elevated plasminogen activator inhibitor-1 (PAI-1), a serine protease inhibitor, represents a risk factor for thrombosis and atherosclerosis [44], we set out to investigate its concentrations in SAA-stimulated HCAEC in the presence/absence of drugs. PAI-1 secretion, as measured by ELISA in cell culture supernatants, was significantly increased in SAA-treated HCAEC in the presence of diclofenac (by 52%), while meloxicam, fluvastatin, etanercept, and certolizumab pegol all significantly decreased its levels (to 71, 73, 57, and 58%, resp.) (Figure 3).

**Figure 4:**
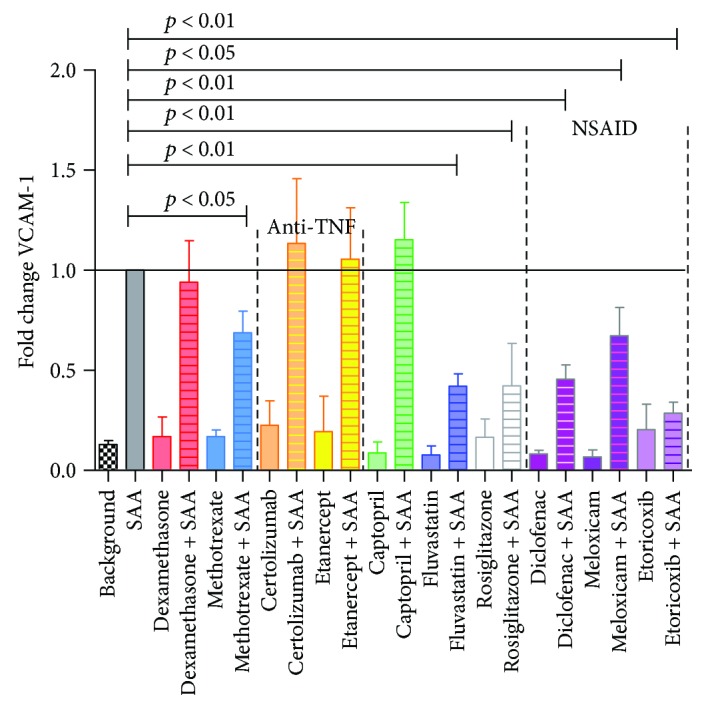


In order to examine the effects of drugs on the SAA-stimulated adhesion molecule, sVCAM-1 in HCAEC, ELISA was performed. Soluble VCAM-1 levels in HCAEC supernatants were significantly inhibited by methotrexate (to 69%), by fluvastatin and rosiglitazone (both to 42%), by diclofenac (to 46%), by meloxicam (to 67%), and most potently by etoricoxib (to 29%), while neither of the TNF inhibitors significantly changed sVCAM-1 levels (Figure 4).

**Figure 5:**
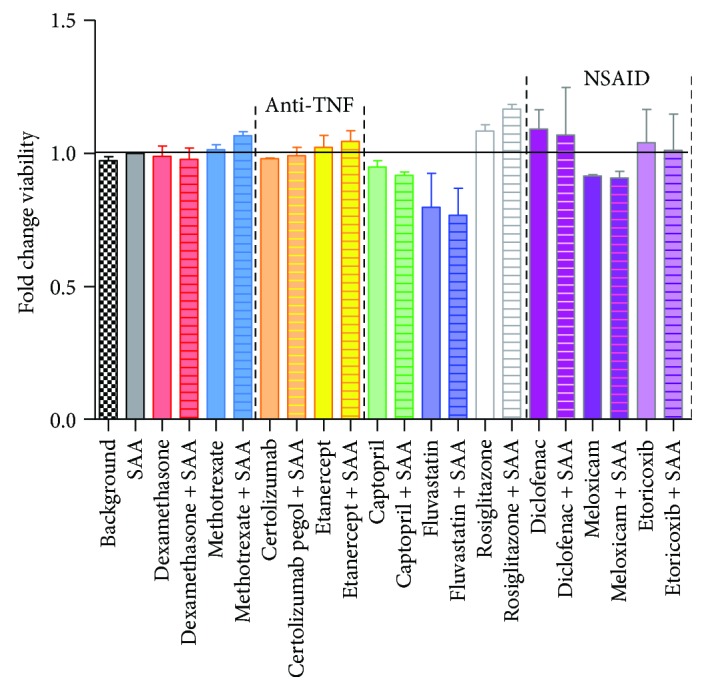


To determine the effects of SAA treatment in the presence/absence of drugs on HCAEC viability, proliferation was assessed based on tetrazolium reduction. No significant changes in absorbance were observed after treatment of HCAEC with drugs alone or in combination with SAA, with respect to the untreated cells (Figure 5).

## 4. Discussion

HCAEC have previously been shown to exhibit increased responsiveness to inflammation and coagulation compared to HUVEC or human microvascular endothelial cells (HMVEC), which could account for greater susceptibility of coronary arteries to inflammation and atherogenesis leading to CV pathology [18]. SAA has previously been reported to play a causal role in atherogenesis in animal and human studies [45]; however, the role of drugs in SAA-stimulated HCAEC has not been investigated till now. Thus, HCAEC represent an optimal cellular model system for evaluating drug effectiveness in an elevated SAA milieu, mimicking *in vivo* activated endothelium.

No drugs applied alone to HCAEC, in our study, exhibited significantly changed levels of tested parameters, including viability, with respect to the untreated cells.

Interestingly, the most effective drug in the presence of SAA was fluvastatin, with the greatest inhibition of all parameters tested, specifically IL-8, VCAM-1, IL-6, and PAI-1 (Figures 1–4). Fluvastatin was reported to induce eNOS, as well as NO and prostaglandin I_2_ production in HUVEC and in human aortic endothelial cells within the first 24 h. In the next 24 h, statins also induced COX-1 and prostacyclin synthase expression [46]. The biphasic effect in vasodilatation is presumably potentiated, as researchers found that eNOS activation leads to iNOS and nitrosylation of COX-2 [39, 47]. Nitrosylated COX-2 produces epi-lipoxin A_4_ (epi-LXA_4_), a potent anti-inflammatory mediator and competitor ligand of SAA for their common LXA_4_ receptor, ALX/FPR2 [48, 49]. Numerous studies on fluvastatin showed, in addition to LDL modification and endothelial function, also effects on smooth muscle cell proliferation, immunomodulation, plaque stabilization, and antithrombotic activity [50]. In HUVEC, multiple studies showed that fluvastatin inhibited CRP-induced TNF*α* expression and NF-*κ*B activation [51], as well as attenuated PAI, tPA [52], and endothelin, while increasing prostacyclin [53]. Inoue et al. [54] reported on fluvastatin reducing IL-6, IL-1*β*, COX-1, and COX-2 and increasing PPAR*α* and PPAR*γ*, in response to different stimuli (specifically lipopolysaccharides, phorbol 12-myristate 13-acetate, and TNF*α*). However, the current study is the first to our knowledge, showing marked decrease of IL-6, IL-8, VCAM-1, and PAI-1 following fluvastatin application to HCAEC, in combination with SAA. In a rabbit model, fluvastatin was reported to reduce TF expression and content of macrophages at atherosclerotic lesions [55]. Fluvastatin has pleiotropic, anti-inflammatory, and antiatherogenic effects including suppression of leukocyte cytokine release, reduction in ROS, amelioration of platelet hyperreactivity, and smooth muscle cell proliferation [6, 56]. Statins prevent oxidative stress and increase vascular nitric oxide (NO) production, so even acute use with intravenous application has been suggested [57]. One fact leading to suppressive effects in inflammatory processes is that by inhibiting mevalonate synthesis, isoprenylation of small GTP-binding proteins is also inhibited, which is required for maintaining NADPH oxidase activity [58] and Ras-like proteins (Rho, Rac). Important for improving endothelial function is that statins induce eNOS through various mechanisms and eNOS-deficient mice are resistant to statin-mediated cardioprotection, mainly due to limiting adherence and leukocyte accumulation [35, 56].

Methotrexate also lowered the effects of SAA on inflammatory cytokines IL-6, IL-8, and sVCAM-1 in HCAEC. We used a final concentration of 1 *μ*M, as doses 0.1–1 *μ*M represent levels achieved in vivo with a low-dose regimen [59]. MTX is known to significantly reduce risk of CV disease in RA and, in contrast to COX-2 inhibitors, demonstrate also atheroprotective properties [60–62]. Besides the reported improvement of systemic autoimmune patient lipid profile [6], Yamasaki et al. [63] found decreased ICAM and VCAM expression with MTX treatment in HUVEC, which was confirmed by Johnston et al. who showed that MTX anti-inflammatory action is predominantly due to suppression of adhesion molecules (e.g., ICAM and cutaneous lymphocyte antigen) through adenosine-mediated or polyglutamate MTX [64]. MTX also decreased AS lesion size, inhibited macrophage migration, and lowered TNF*α*-stimulated HUVEC expression of proinflammatory cytokines (e.g., TNF*α*, vascular adhesion protein 1, IL-1*β*, CXCL2, and TLR2) [65].

While dexamethasone **(**5 *μ*M) did not affect the levels of proinflammatory cytokines or adhesion molecules in our HCAEC model system, EULAR recommendations promote dexamethasone in early arthritis, at doses 7–10 mg/day for less than 6 months [6], while long-term standard therapy is suggested for other rheumatic diseases, such as giant cell arteritis. Dexamethasone reduced IL-6, IL-8, and PGE_2_ induced by IL-1*β* in osteoarthritic and RA fibroblasts [66]; reduced IL-6 and only minor IL-8 in HUVEC in response to TNF*α* [67]; and decreased constitutive MCP-1, but not induced MCP-1 by TNF*α* [68]. Surprisingly, researchers found connections with thromboembolic events and acceleration of inflammation during inflammatory disease states in long-term GC use with increased acute myocardial infarction and CV events [6, 69–71]. This could be due, in part, to nonresponse of I*κ*B transcription to dexamethasone in endothelial cells, contrary to HeLa and THP-1, where I*κ*B is increased under GC thereby suppressing NF-*κ*B [72]. High-dose (1 mM) dexamethasone primed HUVEC for higher expression of adhesion molecules (VCAM, ICAM, and E-selectin) enhancing neutrophil migration, as well as coagulation/fibrinolysis with increased expression of vWf, PAI-1, and tissue factor [73].

In our assays, we used two different anti-TNF*α* biological drugs, specifically etanercept (soluble TNFR2 fusion protein with Fc fragment of human IgG) and certolizumab (human Fab fragment binding TNF*α* with attached pegol to improve pharmacokinetics). Both showed significant decreases in only PAI-1 in our SAA-treated HCAEC (Figure 3), while not exerting major effects on proinflammatory IL-6 or IL-8. Data suggest that neutralizing soluble TNF*α* is not sufficient to attenuate gastrointestinal Crohn's disease [74, 75]. The influence of TNF*α* inhibitors on CV events in RA patients is still elusive, since many studies on larger sample sizes report different results, but an overall trend to reduce CV disease is indicated [76, 77].

We have previously tested for detection of released levels of TNF*α* from SAA-stimulated HCAEC and found them to be very low [78]. That is why TNF*α* has not been included in the compilation of tested molecules, for example, IL-6, IL-8, PAI-1, and VCAM-1, in this study. TNF*α* itself had been previously tested as a single inducer of HCAEC and was shown to upregulate GRO*α*, IL-6, IL-8, and MCP-1 [79]. Consequently, it would be of further interest to determine the effects of drugs, such as anti-TNF*α* inhibitors, methotrexate, and steroids on TNF*α*-activated HCAEC. In such a model, one might speculate that besides etanercept acting to block circulating TNF*α* levels, another hypothetical mode of action could, in part, also come from etanercept binding to the transmembrane form of TNF*α* [75], which could be tested for.

Captopril did not act inhibitory for any of the tested molecules in HCAEC, with only an increased effect on IL-6 observed. Protection of bovine endothelial cells against oxidative stress-induced apoptosis was shown with captopril [80], while reduced ROS, glutathione (GSH) consumption, and inhibition of NF-*κ*B activation were observed with the ACE inhibitor zofenoprilat in HUVEC [81]. There was a short-term antioxidant suppressive effect on redox-sensitive NF-*κ*B activation with captopril reported in sarcoma cells [82], while a long-term role in activating NF-*κ*B and transcription of only certain, protective proteins was suggested, such as manganese superoxide dismutase [83]. Captopril was shown to increase prostacyclin and reduce PAI-1 in porcine aortic endothelial cells and smooth muscle cells [84, 85].

Many beneficial effects were suggested for PPAR*γ* activity, starting with influencing endothelial dysfunction [86, 87]. PPAR*γ* is constitutively active in endothelial cells, suppressing adhesive molecules [88] and cytokine/chemokine expression caused by NF-*κ*B and AP-1 activation. TZDs have been shown to reduce superoxide generation and inhibit expression of VCAM-1, ICAM-1, and lectin-like oxidized LDL receptor and hence inhibit inflammation of endothelial cells [89–92], suggesting an important role of endothelial PPAR*γ* in the development of AS. Our results confirm the data by Xin et al. [93] who reported that a PPAR*γ* agonist (in our case, rosiglitazone in the presence/absence of SAA) increased PAI-1 above background, and further reports indicate decreased levels of VCAM-1 in HUVEC [86, 92], whereby we also show attenuated levels of IL-6 in HCAEC.

All NSAIDs, diclofenac, meloxicam, and etoricoxib, significantly lowered the adhesion molecule VCAM-1 in SAA-treated HCAEC as compared to untreated (Figure 4). Besides this beneficial effect, we observe that diclofenac increased PAI-1, while meloxicam elevated IL-8. Etoricoxib was the only NSAID used in our study to lower both proinflammatory IL-6 and IL-8, while meloxicam was the only NSAID significantly lowering PAI-1 in SAA-stimulated HCAEC (Figures 1–3).

Few studies have been published on the effects of NSAIDs at the cellular levels, making direct comparisons difficult. When etoricoxib was administered preoperatively to patients requiring hip replacement surgery, there was a significant reduction in IL-6 levels in patient plasma observed, with better pain relief, after the surgery [94], which together with our study indicates that etoricoxib could be the NSAID of choice, for lowering proinflammatory cytokines, such as IL-6 and IL-8. Rainsford et al. [95] reported on the effects of meloxicam on human and porcine cartilage explants, as well as human synovial tissue explants. They observed that meloxicam did not affect synovial production of the proinflammatory IL-1 or IL-8 but significantly increased IL-6. This is closer to our study, which otherwise shows an elevation in IL-8 but unchanged IL-6 in HCAEC. Chu et al. [96] reported that meloxicam suppressed PAI-1 secretion from *ex vivo* cultured human osteoarthritic cartilage, meniscus, and synovium at 48 h, similarly, as we currently report for HCAEC at 24 h. However, as the 2010 review on diclofenac showed [42] the modalities of action of NSAIDs could extend well beyond COX inhibition, to further modulate substrate P, peroxisome proliferator activated receptor *γ*, acid sensing ion channels, and nitric oxide-cGMP antinociceptive pathway, among others.

The reason for the specific responsiveness of HCAEC to different drugs, contrary to other types of cells, could be that the endothelium of arteries (versus veins) exhibits (a) specific and intrinsic expression patterns and unique response profiles leading to inflammation and atherosclerosis and (b) greater susceptibility of HCAEC to inflammatory stimuli, specifically pathological concentrations of SAA and IL-1*β*, as opposed to HUVEC and HMVEC [17, 18].

However, our model has some more or less obvious limitations. One limitation related to this cellular HCAEC experimental model is, at the same time, its benefit, namely that HCAEC are primary endothelial cells of the coronary artery, taken from the human body and expanded *ex vivo* and cultured *in vitro.* Thus, they represent a nonsynchronous population of cells and a more optimal model closely mimicking the situation in coronary arteries, as opposed to cell lines, which would otherwise give more homogeneous results, but would be further from the *in vivo* situation. Furthermore, our HCAEC model portrays the limitation of looking at a single inducer (e.g., acute phase SAA), which never occurs *in vivo*; however, a chronically elevated acute phase response, even one conveying low-grade inflammation, is a threat to the coronary arteries and early development of cardiovascular diseases. Clear limitations of the current experimental HCAEC model are that tissue remodelling or vascular aging important in the development of atherosclerosis cannot be addressed, nor the effects of lifestyle changes, such as diet and/or exercise. On the other hand, a cellular model enables rapid screening for drug candidates, restricts the necessary number of animal experiments, and allows for an unlimited access to cells. Taken together, the marked and differential influence of the tested medications on SAA-activated HCAEC could be important for controlling atherogenesis in RA patients. In addition to the well-known protective effects of methotrexate, confirmed by the current study (e.g., lowering of IL-6, IL-8, and VCAM-1), there was a lack of response observed with anti-TNF*α* inhibitors, presumably due to the fact that SAA itself does not induce TNF*α* in HCAEC [78]. In regard to the lack of response of SAA-treated HCAEC to dexamethasone, there could be several considerations: (a) hydrocortisone is present in the endothelial cell medium, which could already mask some of the effects; (b) dexamethasone actually enhances inflammatory responses in ATP-induced endothelial cells [97], and high-dose dexamethasone sensitizes HUVEC to the effect of inflammatory mediators and induces a proadhesive environment [73]; (c) dexamethasone exerted limited effects on TNF*α*- or IL-1*β*-treated HUVEC at 24 h on the gene expression of IL-6, IL-8, and VCAM-1 [98], similar to our model; and (d) long-term use of glucocorticoids increased the rate of acute myocardial infarction and cardiovascular events [6]. One explanation is that dexamethasone does not increase I*κ*B*α* in endothelial cells, as it does in other cell types, such as monocytes and lymphocytes [72], providing a mechanism of why dexamethasone does not inhibit inflammatory responses in HCAEC.

Finally, we emphasize the beneficial role of fluvastatin in our model of primary human coronary artery endothelial cells. It is interesting to speculate whether the beneficial effects in HCAEC of fluvastatin could be the consequence of epi-lipoxin A_4_, a potent anti-inflammatory mediator, produced from the nitrosylated COX-2 (in absence of acetylation) via iNOS and eNOS [39].

In the future, more data on patients already taking fluvastatin could be beneficial, in order to determine possible effects in preventing premature atherosclerosis and CV disease in RA.

## References

[1] Shoenfeld Y., Gerli R., Doria A. (2005). Accelerated atherosclerosis in autoimmune rheumatic diseases. *Circulation*.

[2] Sherer Y., Shoenfeld Y. (2006). Mechanisms of disease: atherosclerosis in autoimmune diseases. *Nature Clinical Practice Rheumatology*.

[3] Bijl M. (2003). Endothelial activation, endothelial dysfunction and premature atherosclerosis in systemic autoimmune diseases. *The Netherlands Journal of Medicine*.

[4] Matsuura E., Kobayashi K., Lopez L. R. (2009). Atherosclerosis in autoimmune diseases. *Current Rheumatology Reports*.

[5] Nurmohamed M. T. (2014). Cardiovascular risk in rheumatoid arthritis: when does it really start?. *Expert Review of Cardiovascular Therapy*.

[6] Atzeni F., Turiel M., Caporali R. (2010). The effect of pharmacological therapy on the cardiovascular system of patients with systemic rheumatic diseases. *Autoimmunity Reviews*.

[7] Lauper K., Gabay C. (2017). Cardiovascular risk in patients with rheumatoid arthritis. *Seminars in Immunopathology*.

[8] Choy E., Ganeshalingam K., Semb A. G., Szekanecz Z., Nurmohamed M. (2014). Cardiovascular risk in rheumatoid arthritis: recent advances in the understanding of the pivotal role of inflammation, risk predictors and the impact of treatment. *Rheumatology*.

[9] Doria A., Sherer Y., Meroni P. L., Shoenfeld Y. (2005). Inflammation and accelerated atherosclerosis: basic mechanisms. *Rheumatic Diseases Clinics of North America*.

[10] Ridker P. M. (1999). Inflammation, atherosclerosis, and cardiovascular risk: an epidemiologic view. *Blood Coagulation & Fibrinolysis: An International Journal in Haemostasis and Thrombosis*.

[11] Poredos P., Jezovnik M. K. (2016). The role of inflammatory biomarkers in the detection and therapy of atherosclerotic disease. *Current Vascular Pharmacology*.

[12] Cunnane G., Grehan S., Geoghegan S. (2000). Serum amyloid A in the assessment of early inflammatory arthritis. *The Journal of Rheumatology*.

[13] Johnson B. D., Kip K. E., Marroquin O. C. (2004). Serum amyloid A as a predictor of coronary artery disease and cardiovascular outcome in women: the National Heart, Lung, and Blood Institute-sponsored Women's Ischemia Syndrome Evaluation (WISE). *Circulation*.

[14] Morrow D. A., Rifai N., Antman E. M. (2000). Serum amyloid A predicts early mortality in acute coronary syndromes: a TIMI 11A substudy. *Journal of the American College of Cardiology*.

[15] King V. L., Thompson J., Tannock L. R. (2011). Serum amyloid A in atherosclerosis. *Current Opinion in Lipidology*.

[16] Zhao Y., He X., Shi X. (2010). Association between serum amyloid A and obesity: a meta-analysis and systematic review. *Inflammation research: official journal of the European Histamine Research Society [et al]*.

[17] Lakota K., Mrak-Poljšak K., Rozman B., Kveder T., Tomšič M., Sodin-Semrl S. (2007). Serum amyloid A activation of inflammatory and adhesion molecules in human coronary artery and umbilical vein endothelial cells. *European Journal of Inflammation*.

[18] Lakota K., Mrak-Poljsak K., Rozman B., Sodin-Semrl S. (2009). Increased responsiveness of human coronary artery endothelial cells in inflammation and coagulation. *Mediators of Inflammation*.

[19] Eggert M., Schulz M., Neeck G. (2001). Molecular mechanisms of glucocorticoid action in rheumatic autoimmune diseases. *The Journal of Steroid Biochemistry and Molecular Biology*.

[20] De Bosscher K., Vanden Berghe W., Haegeman G. (2003). The interplay between the glucocorticoid receptor and nuclear factor-*κ*B or activator protein-1: molecular mechanisms for gene repression. *Endocrine Reviews*.

[21] Chen D. Y., Chih H. M., Lan J. L., Chang H. Y., Chen W. W., Chiang E. P. (2011). Blood lipid profiles and peripheral blood mononuclear cell cholesterol metabolism gene expression in patients with and without methotrexate treatment. *BMC Medicine*.

[22] Hobl E. L., Mader R. M., Erlacher L. (2011). The influence of methotrexate on the gene expression of the pro-inflammatory cytokine IL-12A in the therapy of rheumatoid arthritis. *Clinical and Experimental Rheumatology*.

[23] Paleolog E. (1997). Target effector role of vascular endothelium in the inflammatory response: insights from the clinical trial of anti-TNF alpha antibody in rheumatoid arthritis. *Molecular Pathology*.

[24] Grattendick K. J., Nakashima J. M., Feng L., Giri S. N., Margolin S. B. (2008). Effects of three anti-TNF-*α* drugs: etanercept, infliximab and pirfenidone on release of TNF-*α* in medium and TNF-*α* associated with the cell in vitro. *International Immunopharmacology*.

[25] Scallon B., Cai A., Solowski N. (2002). Binding and functional comparisons of two types of tumor necrosis factor antagonists. *The Journal of Pharmacology and Experimental Therapeutics*.

[26] Elliott M. J., Maini R. N., Feldmann M. (1994). Repeated therapy with monoclonal antibody to tumour necrosis factor *α* (cA2) in patients with rheumatoid arthritis. *Lancet*.

[27] Erdos E. G. (1976). Conversion of angiotensin I to angiotensin II. *The American Journal of Medicine*.

[28] Zhuo J., Moeller I., Jenkins T. (1998). Mapping tissue angiotensin-converting enzyme and angiotensin AT1, AT2 and AT4 receptors. *Journal of Hypertension*.

[29] Li D., Singh R. M., Liu L. (2003). Oxidized-LDL through LOX-1 increases the expression of angiotensin converting enzyme in human coronary artery endothelial cells. *Cardiovascular Research*.

[30] Tom B., Dendorfer A., de Vries R., Saxena P. R., Jan Danser A. H. (2002). Bradykinin potentiation by ACE inhibitors: a matter of metabolism. *British Journal of Pharmacology*.

[31] Nowak W., Errasti A. E., Armesto A. R., Santin Velazque N. L., Rothlin R. P. (2011). Endothelial angiotensin-converting enzyme and neutral endopeptidase in isolated human umbilical vein: an effective bradykinin inactivation pathway. *European Journal of Pharmacology*.

[32] Ilieva I., Ohgami K., Jin X. H. (2006). Captopril suppresses inflammation in endotoxin-induced uveitis in rats. *Experimental Eye Research*.

[33] Lowe J. R., Dixon J. S., Guthrie J. A., McWhinney P. (1986). Serum and synovial fluid levels of angiotensin converting enzyme in polyarthritis. *Annals of the Rheumatic Diseases*.

[34] Fortuny J., de Sanjose S., Becker N. (2006). Statin use and risk of lymphoid neoplasms: results from the European case-control study EPILYMPH. *Cancer Epidemiology, Biomarkers & Prevention: A Publication of the American Association for Cancer Research, cosponsored by the American Society of Preventive Oncology*.

[35] Liao J. K. (2004). Statins: potent vascular anti-inflammatory agents. *International Journal of Clinical Practice*.

[36] Ridker P. M., Danielson E., Fonseca F. A. (2008). Rosuvastatin to prevent vascular events in men and women with elevated C-reactive protein. *The New England Journal of Medicine*.

[37] Smith M. E. B., Lee N. J., Haney E., Carson S. (2009). Drug class review: HMG-CoA reductase inhibitors (statins) and fixed-dose combination products containing a statin: final report update 5 [Internet]. https://www.ncbi.nlm.nih.gov/books/NBK47280/?report=classic.

[38] Hetzel J., Balletshofer B., Rittig K. (2005). Rapid effects of rosiglitazone treatment on endothelial function and inflammatory biomarkers. *Arteriosclerosis, Thrombosis, and Vascular Biology*.

[39] Birnbaum Y., Ye Y., Lin Y. (2006). Augmentation of myocardial production of 15-epi-lipoxin-A_4_ by pioglitazone and atorvastatin in the rat. *Circulation*.

[40] Kopp E., Ghosh S. (1994). Inhibition of NF-kappa B by sodium salicylate and aspirin. *Science*.

[41] Staels B., Koenig W., Habib A. (1998). Activation of human aortic smooth-muscle cells is inhibited by PPAR*α* but not by PPAR*γ* activators. *Nature*.

[42] Gan T. J. (2010). Diclofenac: an update on its mechanism of action and safety profile. *Current Medical Research and Opinion*.

[43] Warner T. D., Mitchell J. A. (2008). COX-2 selectivity alone does not define the cardiovascular risks associated with non-steroidal anti-inflammatory drugs. *Lancet*.

[44] Vaughan D. E. (2005). PAI-1 and atherothrombosis. *Journal of Thrombosis and Haemostasis*.

[45] Chait A., Han C. Y., Oram J. F., Heinecke J. W. (2005). Thematic review series: the immune system and atherogenesis. Lipoprotein-associated inflammatory proteins: markers or mediators of cardiovascular disease?. *Journal of Lipid Research*.

[46] Skogastierna C., Luksha L., Kublickiene K., Eliasson E., Rane A., Ekstrom L. (2011). Beneficial vasoactive endothelial effects of fluvastatin: focus on prostacyclin and nitric oxide. *Heart and Vessels*.

[47] Atar S., Ye Y., Lin Y. (2006). Atorvastatin-induced cardioprotection is mediated by increasing inducible nitric oxide synthase and consequent S-nitrosylation of cyclooxygenase-2. *American Journal of Physiology Heart and Circulatory Physiology*.

[48] Sodin-Semrl S., Spagnolo A., Barbaro B., Varga J., Fiore S. (2004). Lipoxin A_4_ counteracts synergistic activation of human fibroblast-like synoviocytes. *International Journal of Immunopathology and Pharmacology*.

[49] El Kebir D., Jozsef L., Filep J. G. (2008). Opposing regulation of neutrophil apoptosis through the formyl peptide receptor-like 1/lipoxin A_4_ receptor: implications for resolution of inflammation. *Journal of Leukocyte Biology*.

[50] Corsini A. (2000). Reviews: Fluvastatin: effects beyond cholesterol lowering. *Journal of Cardiovascular Pharmacology and Therapeutics*.

[51] Wang H. R., Li J. J., Huang C. X., Jiang H. (2005). Fluvastatin inhibits the expression of tumor necrosis factor-+A7E and activation of nuclear factor-+A7o-B in human endothelial cells stimulated by C-reactive protein. *Clinica Chimica Acta*.

[52] Mussoni L., Banfi C., Sironi L., Arpaia M., Tremoli E. (2000). Fluvastatin inhibits basal and stimulated plasminogen activator inhibitor 1, but induces tissue type plasminogen activator in cultured human endothelial cells. *Thrombosis and Haemostasis*.

[53] Seeger H., Mueck A. O., Lippert T. H. (2000). Fluvastatin increases prostacyclin and decreases endothelin production by human umbilical vein endothelial cells. *International Journal of Clinical Pharmacology and Therapeutics*.

[54] Inoue I., Goto S., Mizotani K. (2000). Lipophilic HMG-CoA reductase inhibitor has an anti-inflammatory effect: reduction of MRNA levels for interleukin-1beta, interleukin-6, cyclooxygenase-2, and p22phox by regulation of peroxisome proliferator-activated receptor alpha (PPARalpha) in primary endothelial cells. *Life Sciences*.

[55] Baetta R., Camera M., Comparato C., Altana C., Ezekowitz M. D., Tremoli E. (2002). Fluvastatin reduces tissue factor expression and macrophage accumulation in carotid lesions of cholesterol-fed rabbits in the absence of lipid lowering. *Arteriosclerosis, Thrombosis, and Vascular Biology*.

[56] Jain M. K., Ridker P. M. (2005). Anti-inflammatory effects of statins: clinical evidence and basic mechanisms. *Nature Reviews Drug Discovery*.

[57] Laufs U., Adam O. (2012). Acute effects of statins. *Journal of the American College of Cardiology*.

[58] Rossen R. D. (1997). HMG-CoA reductase inhibitors: a new class of anti-inflammatory drugs?. *Journal of the American College of Cardiology*.

[59] Sinnett M. J., Groff G. D., Raddatz D. A., Franck W. A., Bertino J. S. (1989). Methotrexate pharmacokinetics in patients with rheumatoid arthritis. *The Journal of Rheumatology*.

[60] Westlake S. L., Colebatch A. N., Baird J. (2010). The effect of methotrexate on cardiovascular disease in patients with rheumatoid arthritis: a systematic literature review. *Rheumatology*.

[61] Coomes E., Chan E. S., Reiss A. B. (2011). Methotrexate in atherogenesis and cholesterol metabolism. *Cholesterol*.

[62] Choi H. K., Hernan M. A., Seeger J. D., Robins J. M., Wolfe F. (2002). Methotrexate and mortality in patients with rheumatoid arthritis: a prospective study. *Lancet*.

[63] Yamasaki E., Soma Y., Kawa Y., Mizoguchi M. (2003). Methotrexate inhibits proliferation and regulation of the expression of intercellular adhesion molecule-1 and vascular cell adhesion molecule-1 by cultured human umbilical vein endothelial cells. *The British Journal of Dermatology*.

[64] Johnston A., Gudjonsson J. E., Sigmundsdottir H., Ludviksson B. R., Valdimarsson H. (2005). The anti-inflammatory action of methotrexate is not mediated by lymphocyte apoptosis, but by the suppression of activation and adhesion molecules. *Clinical Immunology*.

[65] Bulgarelli A., Martins Dias A. A., Caramelli B., Maranhao R. C. (2012). Treatment with methotrexate inhibits atherogenesis in cholesterol-fed rabbits. *Journal of Cardiovascular Pharmacology*.

[66] Inoue H., Takamori M., Nagata N. (2001). An investigation of cell proliferation and soluble mediators induced by interleukin 1beta in human synovial fibroblasts: comparative response in osteoarthritis and rheumatoid arthritis. *Inflammation Research*.

[67] Asgeirsdottir S. A., Kok R. J., Everts M., Meijer D. K., Molema G. (2003). Delivery of pharmacologically active dexamethasone into activated endothelial cells by dexamethasone-anti-E-selectin immunoconjugate. *Biochemical Pharmacology*.

[68] Kakizaki Y., Waga S., Sugimoto K. (1995). Production of monocyte chemoattractant protein-1 by bovine glomerular endothelial cells. *Kidney International*.

[69] Suissa S., Bernatsky S., Hudson M. (2006). Antirheumatic drug use and the risk of acute myocardial infarction. *Arthritis and Rheumatism*.

[70] Solomon D. H., Avorn J., Katz J. N. (2006). Immunosuppressive medications and hospitalization for cardiovascular events in patients with rheumatoid arthritis. *Arthritis and Rheumatism*.

[71] Wolfe F., Michaud K. (2008). The risk of myocardial infarction and pharmacologic and nonpharmacologic myocardial infarction predictors in rheumatoid arthritis: a cohort and nested case-control analysis. *Arthritis and Rheumatism*.

[72] Brostjan C., Anrather J., Csizmadia V. (1996). Glucocorticoid-mediated repression of NF*κ*B activity in endothelial cells does not involve induction of I*κ*B*α* synthesis. *The Journal of Biological Chemistry*.

[73] Kerachian M. A., Cournoyer D., Harvey E. J. (2009). Effect of high-dose dexamethasone on endothelial haemostatic gene expression and neutrophil adhesion. *The Journal of Steroid Biochemistry and Molecular Biology*.

[74] Nesbitt A., Fossati G., Bergin M. (2007). Mechanism of action of certolizumab pegol (CDP870): in vitro comparison with other anti-tumor necrosis factor alpha agents. *Inflammatory Bowel Diseases*.

[75] Horiuchi T., Mitoma H., Harashima S., Tsukamoto H., Shimoda T. (2010). Transmembrane TNF-*α*: structure, function and interaction with anti-TNF agents. *Rheumatology*.

[76] Askling J., Dixon W. (2011). Influence of biological agents on cardiovascular disease in rheumatoid arthritis. *Annals of the Rheumatic Diseases*.

[77] Westlake S. L., Colebatch A. N., Baird J. (2011). Tumour necrosis factor antagonists and the risk of cardiovascular disease in patients with rheumatoid arthritis: a systematic literature review. *Rheumatology*.

[78] Lakota K., Mrak-Poljsak K., Bozic B., Tomsic M., Sodin-Semrl S. (2013). Serum amyloid A activation of human coronary artery endothelial cells exhibits a neutrophil promoting molecular profile. *Microvascular Research*.

[79] Artenjak A., Omersel J., Ahlin Grabnar P. (2015). Oxidatively altered IgG with increased immunoreactivity to *β*2-glycoprotein I and its peptide clusters influence human coronary artery endothelial cells. *Lupus*.

[80] Yu W., Akishita M., Xi H. (2006). Angiotensin converting enzyme inhibitor attenuates oxidative stress-induced endothelial cell apoptosis via p38 MAP kinase inhibition. *Clinica chimica acta*.

[81] Cominacini L., Pasini A., Garbin U. (2002). Zofenopril inhibits the expression of adhesion molecules on endothelial cells by reducing reactive oxygen species. *American Journal of Hypertension*.

[82] Murley J. S., Kataoka Y., Cao D., Li J. J., Oberley L. W., Grdina D. J. (2004). Delayed radioprotection by NF*κ*B-mediated induction of Sod2 (MnSOD) in SA-NH tumor cells after exposure to clinically used thiol-containing drugs. *Radiation Research*.

[83] Murley J. S., Kataoka Y., Hallahan D. E., Roberts J. C., Grdina D. J. (2001). Activation of NF*κ*B and MnSOD gene expression by free radical scavengers in human microvascular endothelial cells. *Free Radical Biology & Medicine*.

[84] Xiong Y. L., Zhao H. Y. (1995). Effect of captopril on antithrombus function of endothelium. *Journal of Tongji Medical University*.

[85] Xiong Y. L., Zhao H. Y. (1996). Effect of captopril on proliferation of aortic smooth muscle cells. *Acta Pharmacologica Sinica*.

[86] Jackson S. M., Parhami F., Xi X. P. (1999). Peroxisome proliferator-activated receptor activators target human endothelial cells to inhibit leukocyte-endothelial cell interaction. *Arteriosclerosis, Thrombosis, and Vascular Biology*.

[87] Marx N., Walcher D. (2007). Vascular effects of PPARgamma activators - from bench to bedside. *Progress in Lipid Research*.

[88] Wang N., Verna L., Chen N. G. (2002). Constitutive activation of peroxisome proliferator-activated receptor-gamma suppresses pro-inflammatory adhesion molecules in human vascular endothelial cells. *The Journal of Biological Chemistry*.

[89] Sasaki M., Jordan P., Welbourne T. (2005). Troglitazone, a PPAR-*γ* activator prevents endothelial cell adhesion molecule expression and lymphocyte adhesion mediated by TNF-*α*. *BMC Physiology*.

[90] Imamoto E., Yoshida N., Uchiyama K. (2004). Inhibitory effect of pioglitazone on expression of adhesion molecules on neutrophils and endothelial cells. *BioFactors*.

[91] Mehta J. L., Hu B., Chen J., Li D. (2003). Pioglitazone inhibits LOX-1 expression in human coronary artery endothelial cells by reducing intracellular superoxide radical generation. *Arteriosclerosis, Thrombosis, and Vascular Biology*.

[92] Pasceri V., Wu H. D., Willerson J. T., Yeh E. T. (2000). Modulation of vascular inflammation in vitro and in vivo by peroxisome proliferator-activated receptor-*γ* activators. *Circulation*.

[93] Xin X., Yang S., Kowalski J., Gerritsen M. E. (1999). Peroxisome proliferator-activated receptor gamma ligands are potent inhibitors of angiogenesis in vitro and in vivo. *The Journal of Biological Chemistry*.

[94] Renner B., Walter G., Strauss J., Fromm M. F., Zacher J., Brune K. (2012). Preoperative administration of etoricoxib in patients undergoing hip replacement causes inhibition of inflammatory mediators and pain relief. *European Journal of Pain*.

[95] Rainsford K. D., Ying C., Smith F. C. (1997). Effects of meloxicam, compared with other NSAIDs, on cartilage proteoglycan metabolism, synovial prostaglandin E2, and production of interleukins 1, 6 and 8, in human and porcine explants in organ culture. *The Journal of Pharmacy and Pharmacology*.

[96] Chu S. C., Yang S. F., Lue K. H., Hsieh Y. S., Li T. J., Lu K. H. (2008). Naproxen, meloxicam and methylprednisolone inhibit urokinase plasminogen activator and inhibitor and gelatinases expression during the early stage of osteoarthritis. *Clinica Chimica Acta*.

[97] Ding Y., Gao Z. G., Jacobson K. A., Suffredini A. F. (2010). Dexamethasone enhances ATP-induced inflammatory responses in endothelial cells. *The Journal of Pharmacology and Experimental Therapeutics*.

[98] Kuldo J. M., Westra J., Asgeirsdottir S. A. (2005). Differential effects of NF-*κ*B and p38 MAPK inhibitors and combinations thereof on TNF-*α*- and IL-1*β*-induced proinflammatory status of endothelial cells in vitro. *American Journal of Physiology-Cell Physiology*.

